# Supplementary material for: In Vitro Efficacy of Antibiotic Combinations with Carbapenems and Other Agents against Anaerobic Bacteria
Source: Antibiotics (Basel). 2022 Feb 22;11(3):292. doi: 10.3390/antibiotics11030292 (PMC8944673; doi:10.3390/antibiotics11030292)
Supplement: Supplementary file 1 [file antibiotics-11-00292-s001.zip › antibiotics-1578542-supplementary.pdf]

Supplementary Appendix

This appendix has been provided by the authors to give readers additional information about their work.

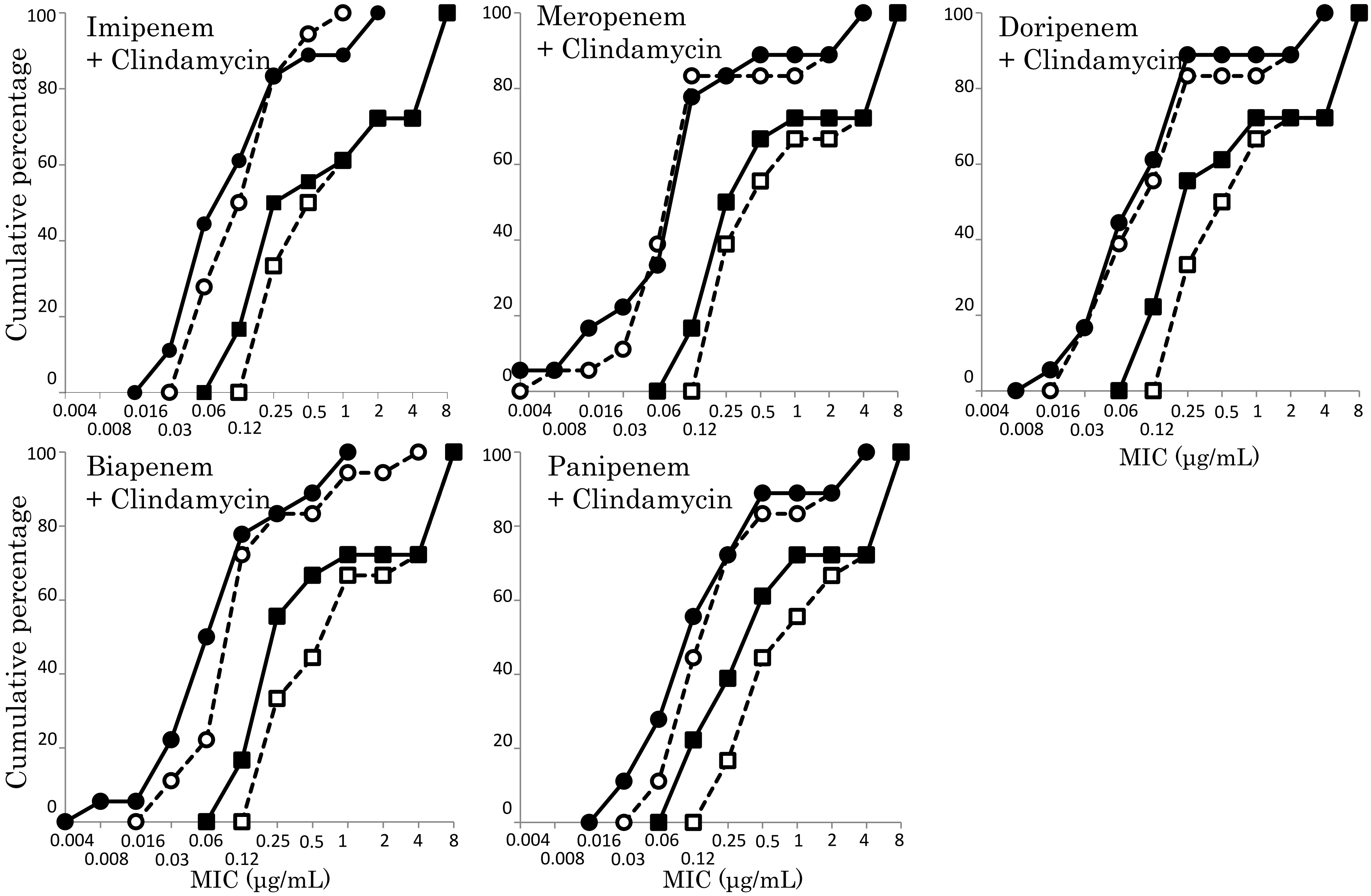

Figure S1. (A) Antibacterial activety of Carbapenems, Clindamycin and combination against *B.fragilis*

Open circle; MIC of Carbapenems, open square; MIC of Clindamycin, closed circle; MIC of Carbapenems combined with Clindamycin, closed square; MIC of Clindamycin combined with Carbapenems.

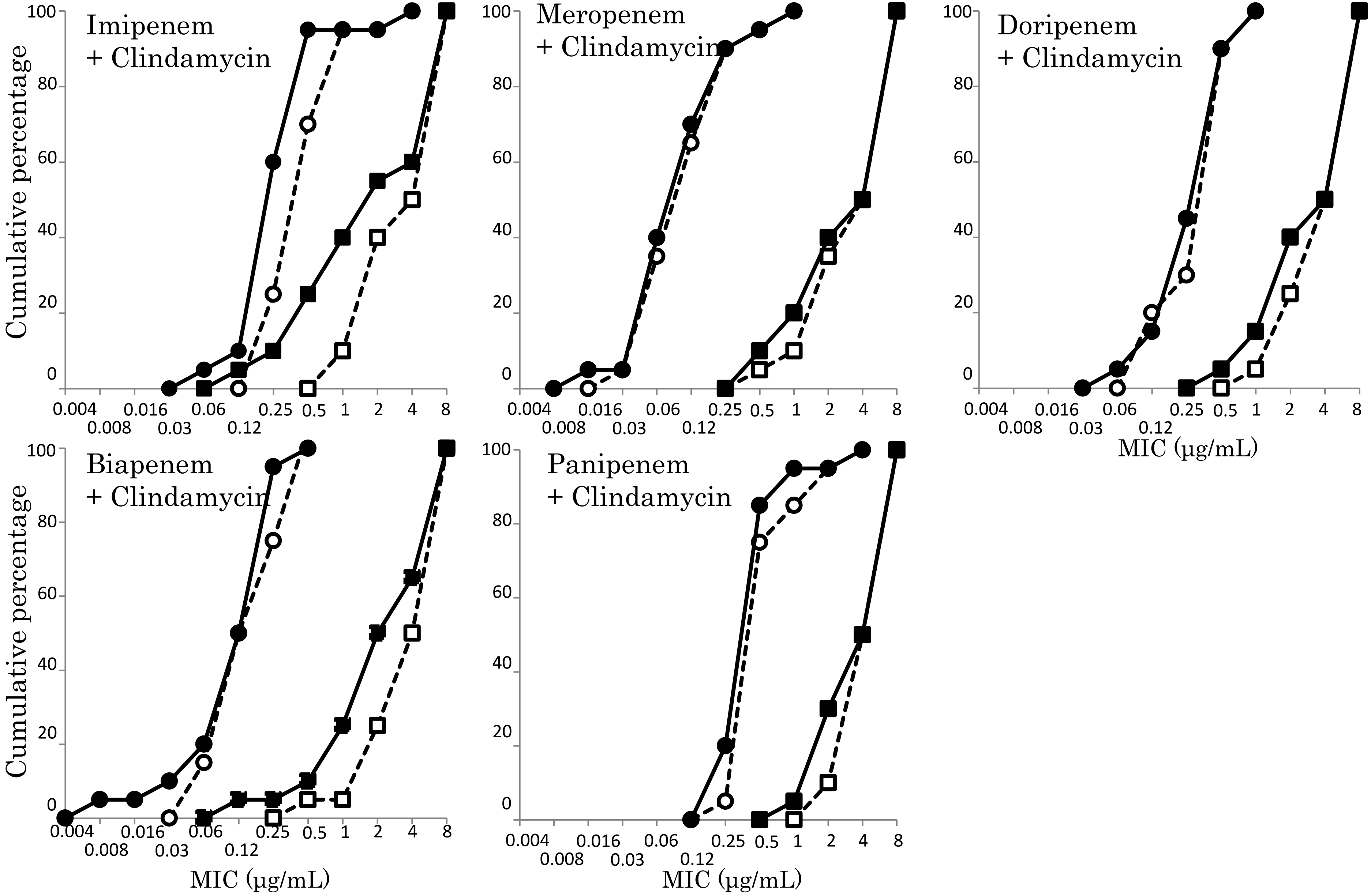

Figure S1. (B) Antibacterial activety of Carbapenems, Clindamycin and combination against *B.thetaiotaomicron*

Open circle; MIC of Carbapenems, open square; MIC of Clindamycin, closed circle; MIC of Carbapenems combined with Clindamycin, closed square; MIC of Clindamycin combined with Carbapenems.

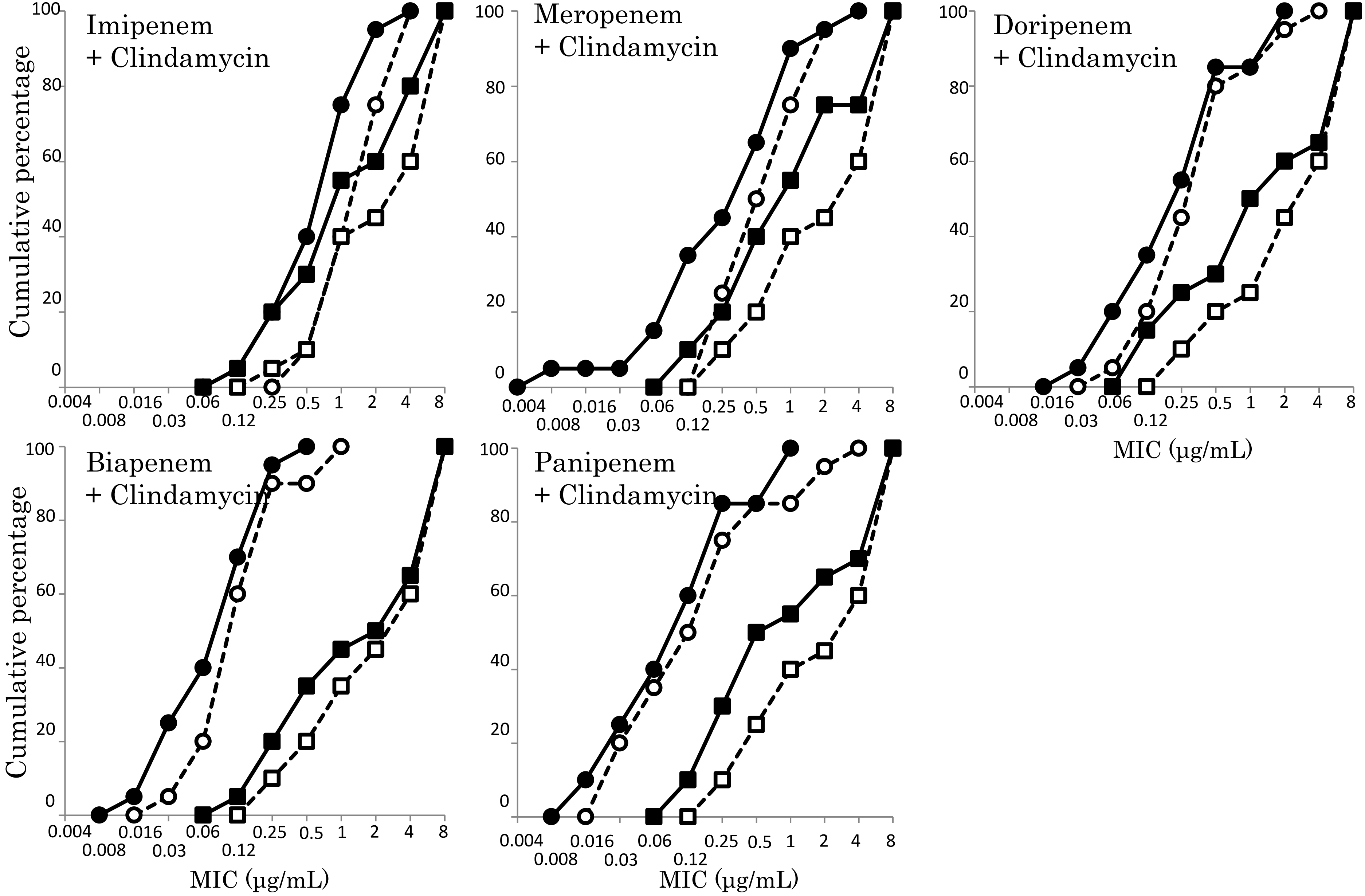

Figure S1. (C) Antibacterial activety of Carbapenems, Clindamycin and combination against *P.distasonis*

Open circle; MIC of Carbapenems, open square; MIC of Clindamycin, closed circle; MIC of Carbapenems combined with Clindamycin, closed square; MIC of Clindamycin combined with Carbapenems.

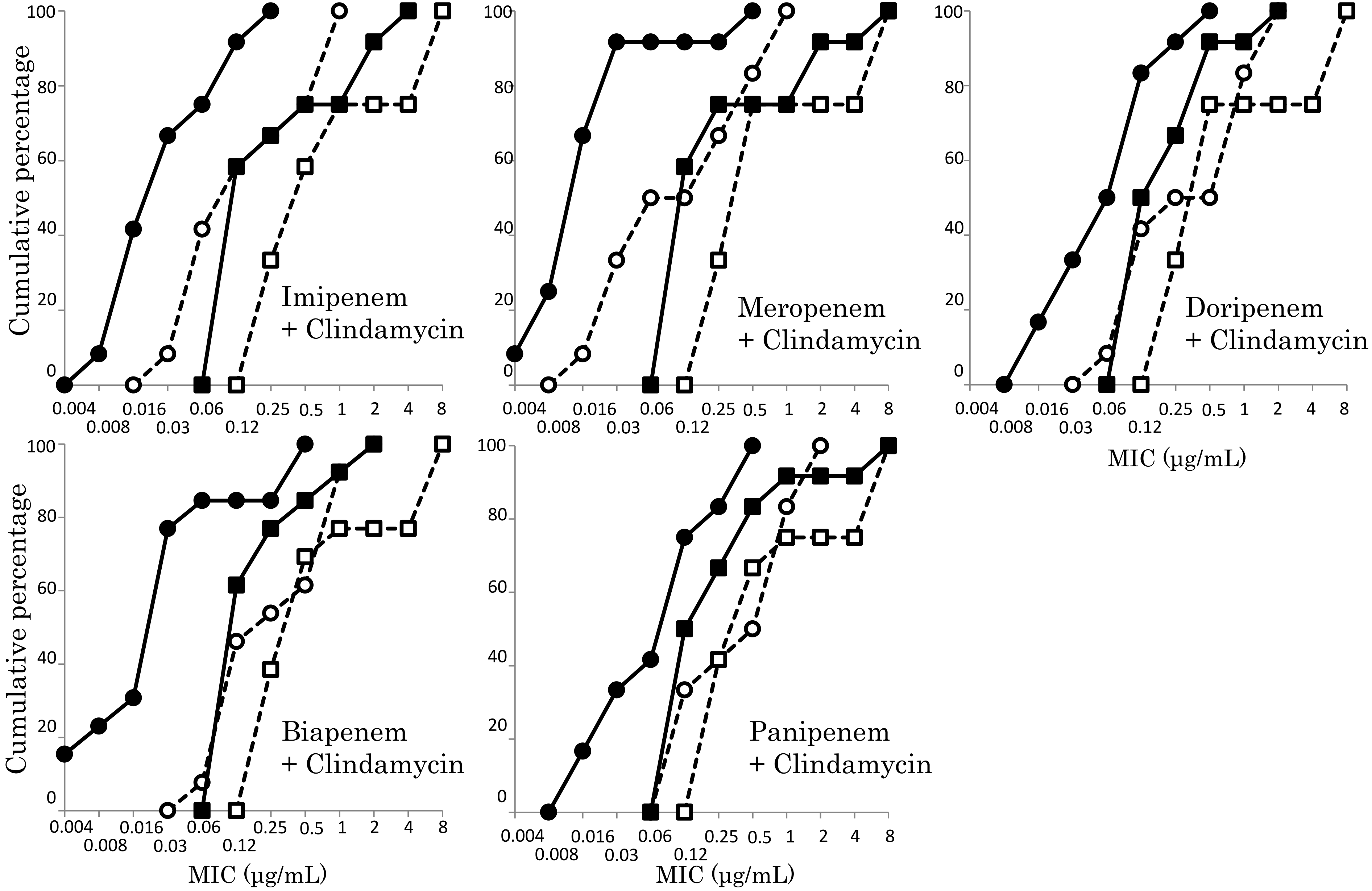

Figure S1. (D) Antibacterial activety of Carbapenems, Clindamycin and combination against *P.anaerobius*

Open circle; MIC of Carbapenems, open square; MIC of Clindamycin, closed circle; MIC of Carbapenems combined with Clindamycin, closed square; MIC of Clindamycin combined with Carbapenems.

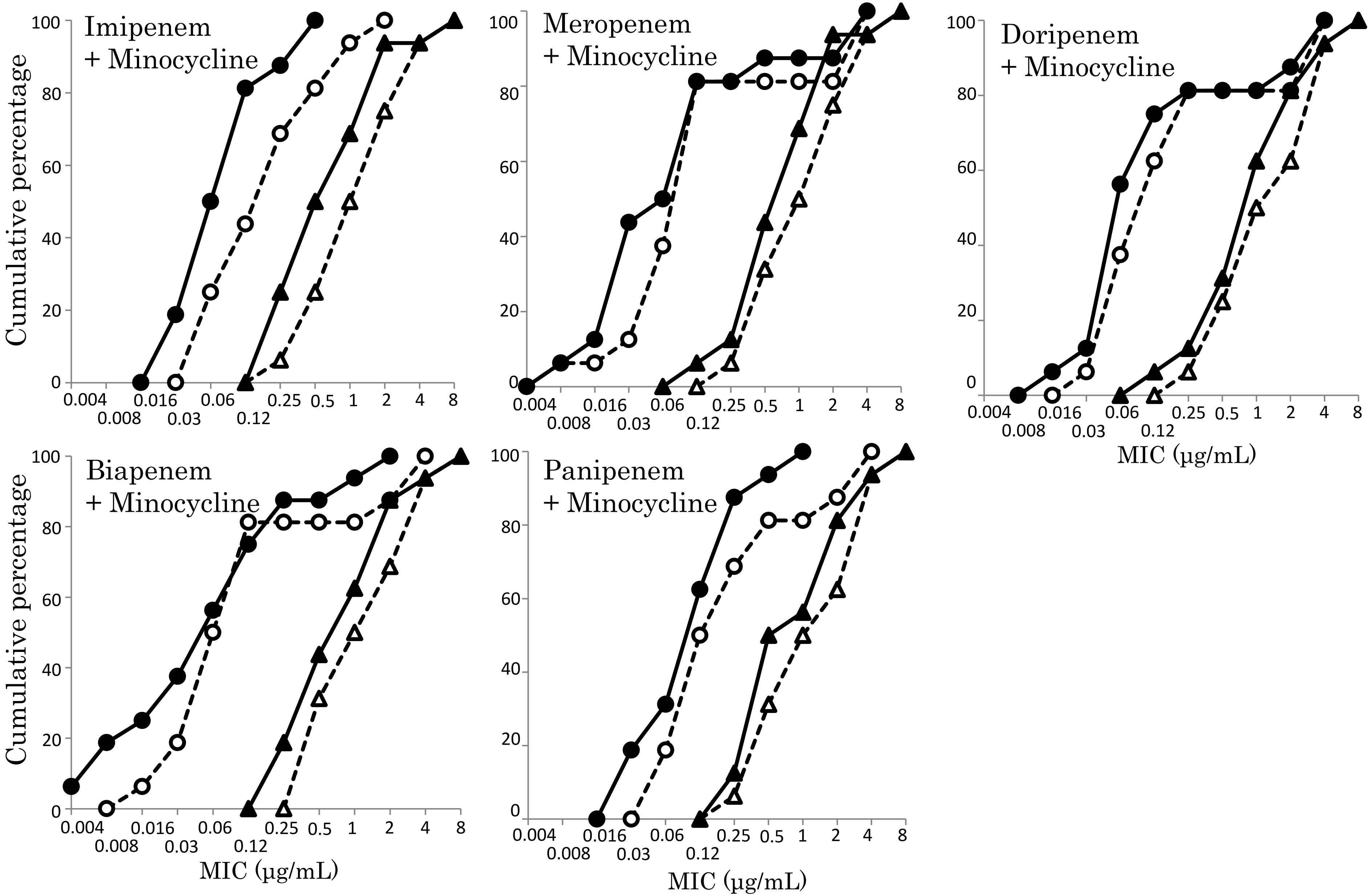

Figure S2. (A) Antibacterial activety of Carbapenems, Minocycline and combination against *B.fragilis*

Open circle; MIC of Carbapenems, open triangle; MIC of Minocycline, closed circle; MIC of Carbapenems combined with Minocycline, closed triangle; MIC of Minocycline combined with Carbapenems.

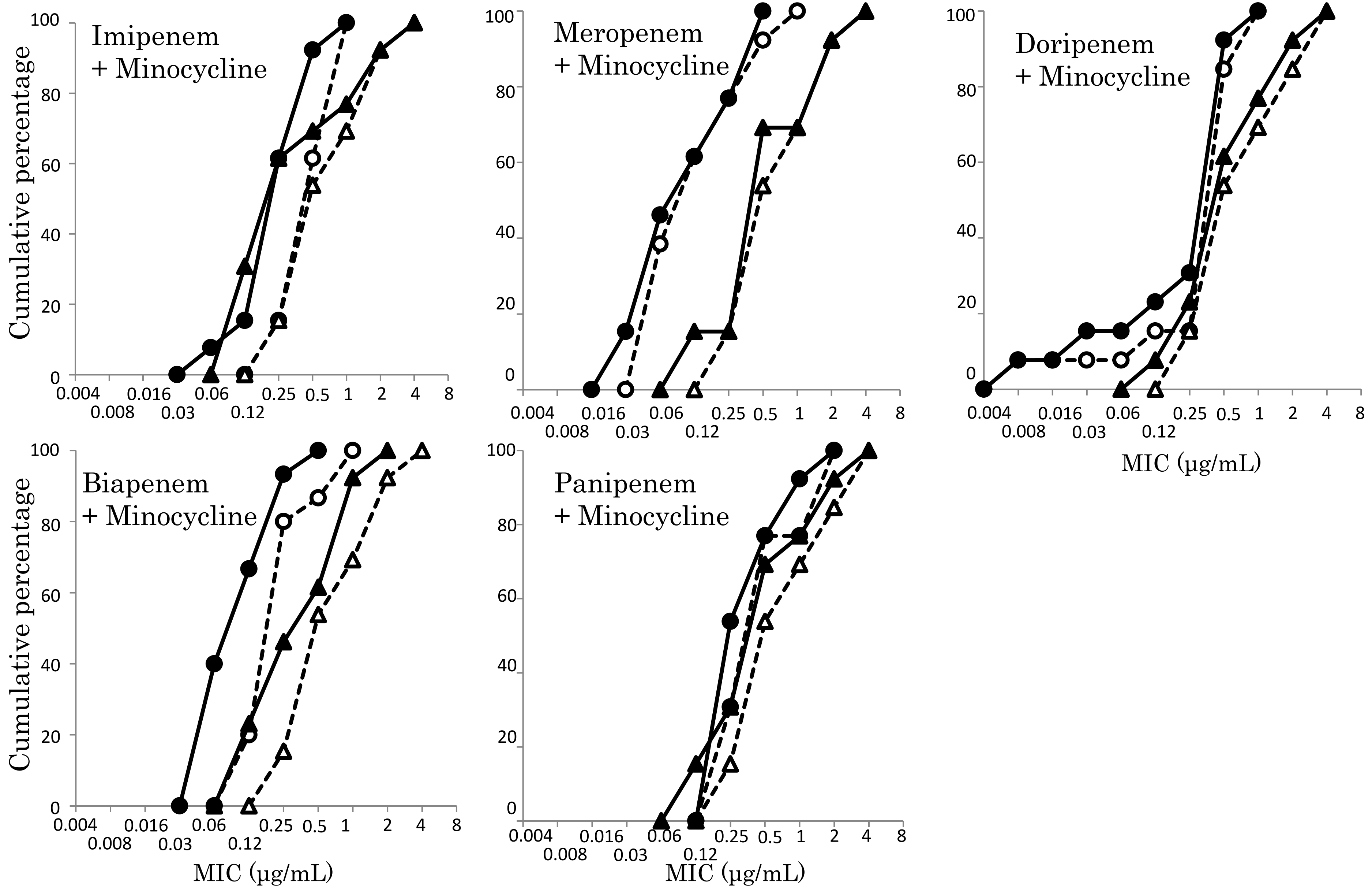

Figure S2. (B) Antibacterial activety of Carbapenems, Minocycline and combination against *B.thetaiotaomicron*

Open circle; MIC of Carbapenems, open triangle; MIC of Minocycline, closed circle; MIC of Carbapenems combined with Minocycline, closed triangle; MIC of Minocycline combined with Carbapenems.

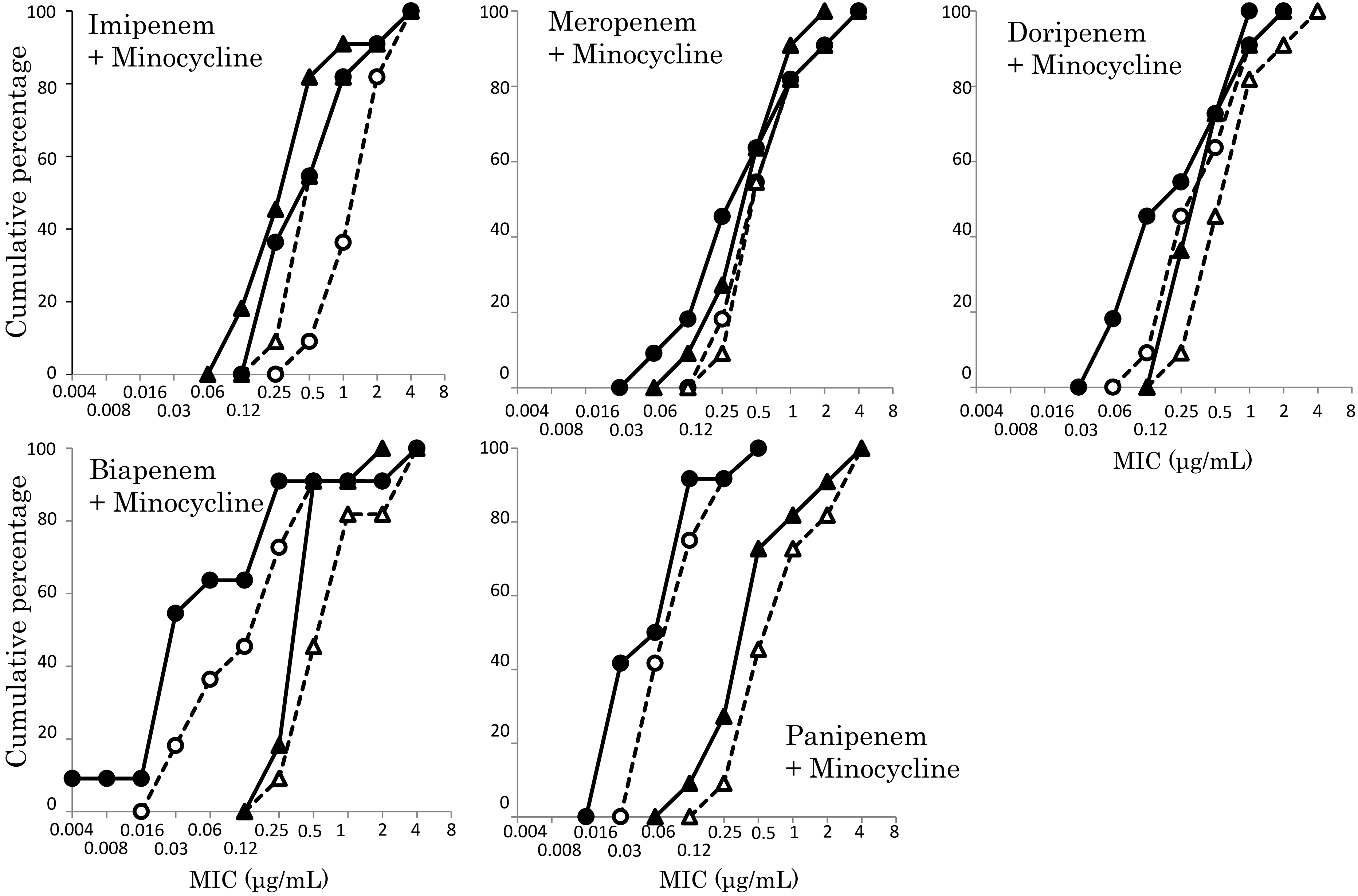

Figure S2. (C) Antibacterial activety of Carbapenems, Minocycline and combination against *B.distasonis*

Open circle; MIC of Carbapenems, open triangle; MIC of Minocycline, closed circle; MIC of Carbapenems combined with Minocycline , closed triangle; MIC of Minocycline combined with Carbapenems.

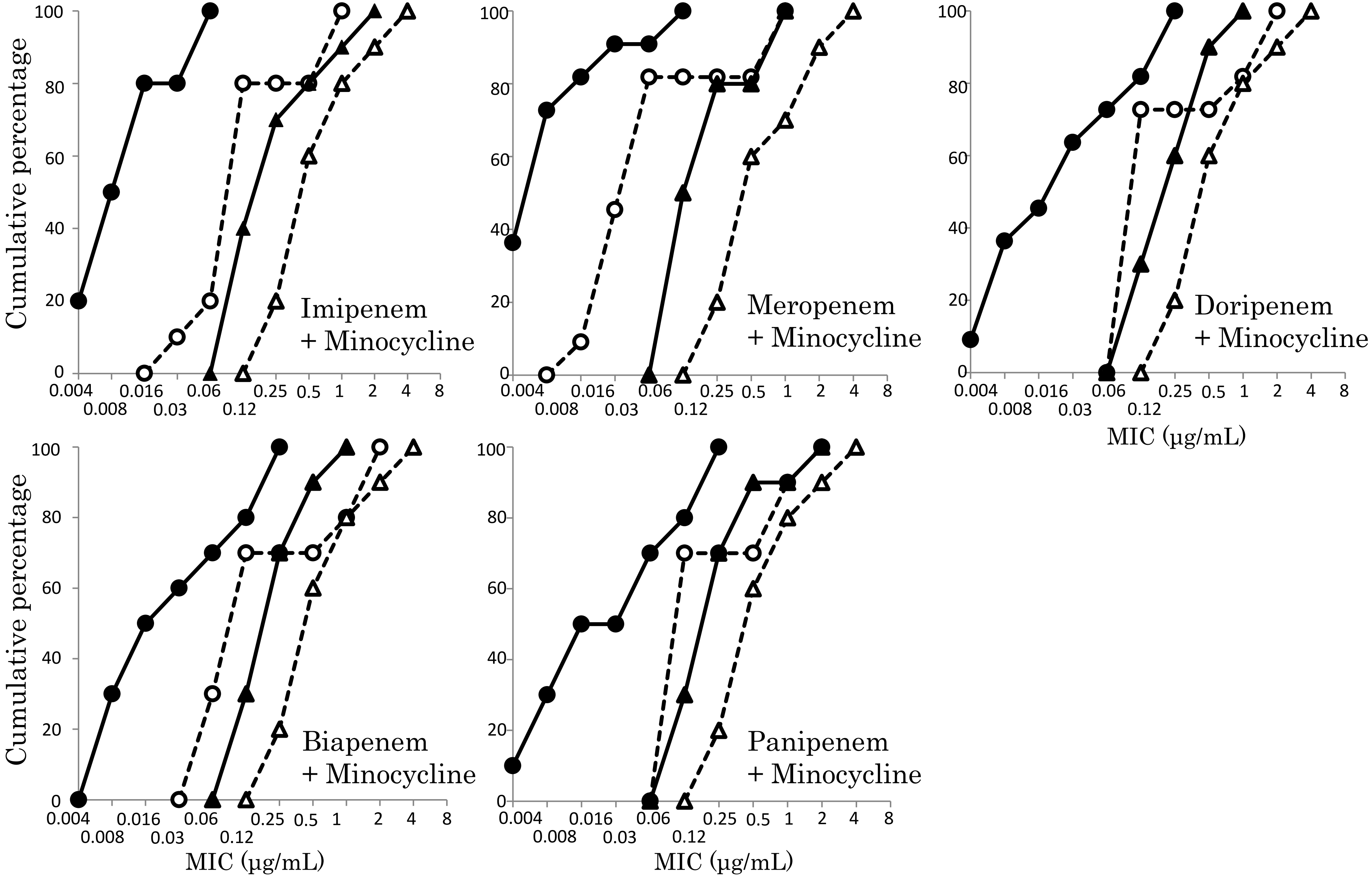

Figure S2. (D) Antibacterial activety of Carbapenems, Minocycline and combination against *P.anaerobius*

Open circle; MIC of Carbapenems, open triangle; MIC of Minocycline, closed circle; MIC of Carbapenems combined with Minocycline , closed triangle; MIC of Minocycline combined with Carbapenems.
